# Is there a social gradient in how youth with mental disorder perform academically? Findings from a Swedish longitudinal register-based study

**DOI:** 10.1186/s12888-021-03448-z

**Published:** 2021-09-06

**Authors:** Evelina Landstedt, Cristian Bortes, Mattias Strandh

**Affiliations:** 1 Department of Social and Psychological Studies, Karlstad University, Universitetsgatan 2, SE-651 88 Karlstad, Sweden; 2 Department of Social Work, Umeå University, SE-907 87 Umeå, Sweden; 3 Centre for Research on Child and Adolescent Mental Health, Karlstad University, SE-651 88 Karlstad, Sweden

## Background

Poor mental health in children and adolescents include anxiety and depressive symptoms, as well as mental disorders [1]. Beyond its detrimental effects on the individual’s quality of life, poor mental health in childhood is a major risk factor for future short- and long-term mental health problems, as well as other adverse social, educational and work-related outcomes [2–6]. Accordingly, it has been shown that self-rated health status in adults is associated with access to health-related and material resources in childhood, and is influenced by (among other things) school factors in youth [7, 8]. Clearly, school is highly important for young people’s general well-being and for establishing future opportunities. Understanding the relationship between mental health and school achievement is key in this regard, particularly given the current deterioration in both mental health and school results in Sweden and worldwide [9–13]. Increasing income inequality in Sweden [14] further calls for research into circumstances early in life that can influence future health as well as social and economic outcomes, for the individual as well as society. Consequently, from an equity perspective, it is important to explore how school-related outcomes are affected when young people are in distress and if these outcomes differ depending on gender and socioeconomic background.

It is well established that academic achievement and other school-related outcomes are associated with mental health status in children and youths [15–17] as well as young university students [18]. However, due to the predominance of cross-sectional studies, the direction of the association is unclear. Nevertheless, based on longitudinal analyses, several models have been proposed in order to understand this relationship. According to the academic-incompetence model, poor academic performance is related to later mental health problems [17, 19]. The adjustment-erosion model, on the other hand, suggests that adolescents with poor mental health perform worse in school than their mentally well peers [5, 17, 20, 21]. Others propose a cascade process of the interplay between internalising and externalising problems in relation to academic difficulties across time [22–24]. A possible contributor to the inconsistencies regarding direction of associations is the vast range of measures applied to reflect different dimensions of poor mental health as well as educational outcomes [23]. Most research also rely on self-reported or parent (or teacher) assessed data on both mental health problems and academic achievement [21, 23, 25–28]. Only a few studies within the field are based on information from registers [29–31]. We argue that it is of value to utilise non-self reported data in exploring the longitudinal relationship between poor mental health in childhood and adolescence, and later academic achievement. Apart from possibly avoiding risk of reporting bias, register data also provide information on more severe mental health problems than is typically investigated in relation to academic achievement, from a large number of individuals.

Although both child/adolescent mental health status and academic achievement are gendered and socially patterned [32–36], there is a knowledge gap relating to gendered and social gradients in the relationship between poor mental health and academic achievement. Some evidence suggests that the association is stronger in girls than in boys [17, 19, 37, 38]. Others argue that associations depend on types of symptoms and age [15, 25]. The role of socioeconomic factors, such as parental level of education and income, is even less explored. The limited evidence available suggest that socioeconomic factors are stronger predictors of academic achievement than various health indicators [39] and that children of low socioeconomic background are disadvantaged both academically and mental health wise [26]. Findings indicate that strong family resources (both cultural and economic) and high neighbourhood socioeconomic status can compensate for the negative impact of mental distress on school achievement [40, 41]. Research on the socioeconomic patterns of social determinants of health and the consequences of (poor) health, suggest that the most privileged would experience fewer and less severe negative educational consequences of prior mental health problems than their less advantaged peers [32, 42, 43].

Accordingly, it is likely that the risk of adverse future outcomes for mentally distressed adolescents differ depending on social background and is greatest among youths from low socioeconomic status (SES) families [44, 45]. However, it is not clear whether mental health status is consistently related to academic achievement across socioeconomic groups. This knowledge gap is especially pronounced in the Swedish context despite the contribution of Brännlund and Edlund (2020), who concluded that family socioeconomic background moderate the relationship between poor mental health in childhood/adolescence and later graduation failure and grade points in upper secondary school students, especially among girls [46].

Systematic reviews of research on emotional problems and school outcomes in children and adolescents conclude that existing evidence to a high degree rely on cross-sectional studies (see e.g. [15, 47]). In order to establish directions of associations, prospective longitudinal designs are needed. Longitudinal research is also important to prevent potential negative or positive affectivity, which would occur if, for example, adolescents with poor mental health were more likely to report poor school performance, or if their healthy peers tend to overrate their achievements [20, 48]. Affectivity can be minimised by examining register data rather than self-reported information. The present study is based on longitudinal register data with yearly observations.

The aim of the present study is to investigate social gradients in the prospective association between childhood/youth mental disorder and academic achievement at age 15–16 in Swedish boys and girls.

## Methods

The study is based on administrative data from the Umeå SIMSAM Lab. Umeå SIMSAM Lab is specifically designed to investigate questions on children’s health and well-being [49]. Data are longitudinal and incorporate medical and social individual-level information from several registers covering the entire Swedish population between 1960 and 2010. Each individual is assigned a unique and fully anonymised personal identification number that links them to family members across registers. In addition to the Total Population Register, this study also used information from other registers as described below. The Regional Ethical Vetting Board in Umeå approved all research based on data from the Umeå SIMSAM Lab, including the present study (Dnr.2010–157-31).

### Population

The population comprises all individuals born between 1990 and 1994 who (according to the Total Population Register) were alive and resided in Sweden in 2010 (*N* = 642,558 boys = 51.38%). These individuals are hereafter referred to as *index persons*. As all data were retrieved from registers, no active consent was given by participants.

### Outcome

Academic achievement is defined as the sum of the index person’s 16 best subject grades during the final year of compulsory schooling (9th grade), when the index persons were 15–16 years old. Data were drawn from the Swedish National Agency for Education’s Pupil Register.

For each subject, a student is assigned a grade ranging from 0 to 20. The summed grade points thus range from 0 to 320 and indicate the child’s general academic achievement (performance, effort, and ambition). This variable was standardised into z-scores (mean = 0, and standard deviation = 1).

### Predictors

In the current study, we use the term ‘mental disorder’ to conceptualise the included indicators of poor mental health: psychiatric hospitalisation events and psychoanaleptic drug prescriptions. These are further described below. Both hospitalisation and medical treatment indicate relatively severe conditions and should therefore neither be conceptualised as, for example, ‘poor mental health’ in a broad sense, nor as the more serious conditions of ‘mental illness’ [1]. Another possible term would have been ‘psychiatric disorder’. However, as the ICD 10 uses the term mental disorders [50], we believe that consistency in conceptualisation is preferred. The term mental disorder has also been used in a recent similar study [51].

*Psychiatric hospitalisations* indicates the number of hospitalisation events due to a main diagnosis of mental disorder according to the International Classification of Disease (ICD-9: 290–319, ICD-10: F00-F99), obtained from the Swedish National Patient Register (NPR) [52]. This variable indicates the total number of hospitalisation events between birth (1990–1994) until the year before the index person received their compulsory school grades (2005–2009). In Sweden, children with mental health problems receive outpatient care at primary care centres or psychiatric specialist care units. Psychiatric hospitalisations occur only in response to acute events (e.g., suicidal events) or if a patient’s health status becomes significantly impaired despite outpatient treatment. Psychiatric hospitalisations therefore corresponds to one psychiatric *inpatient* care event, which indicates severe psychiatric disorder.

*Psychoanaleptic drug prescriptions.* To complement the hospitalisation data and detect individuals with less severe psychiatric health problems, records of prescription drug sales were retrieved from the Swedish Prescribed Drug Register (PDR). These data included details of the patient and the active substance prescribed, classified using the Anatomical Therapeutic Chemical (ATC) Classification System. The information from the PDR was only available to us for the years 2005–2010, when the index persons were 11–16 years of age. The psychoanaleptic drug prescription indicator indicates whether the individual under consideration was prescribed any medication with an ATC classification of N06 (psychoanaleptics) at least once over this 5-year period (no/yes). This category includes Attention-Deficit/Hyperactivity Disorder (ADHD) medications and drugs for sleeping problems and anxiety. Drug prescription has been used as an indicator of poor mental health in similar studies [53].

### Indicators of socioeconomic position

*Parental level of education* is a widely used indicator of family socioeconomic position (SEP) [54], especially in studies on academic achievement [55, 56]. In this study, *parental level of education* was operationalised as the highest level of education attained by either parent when the child was 7 years old. Prior to 2018, children in Sweden started school (year 1) at age 7 years (after 2018, compulsory schooling start at age 6). The indicator is based on the status of the index person’s parent (either mother or father) with the highest education level. Four levels were defined: (1) compulsory school (1st-9th grade); (2) 2 years’ upper secondary education; (3) 3 years’ upper secondary education; and, (4) post-secondary education. These four groups correspond to the existing levels of education in Sweden when the parents attended upper secondary school. A 2 year upper secondary education includes vocational training whereas 3 years of upper secondary education qualifies for tertiary studies. Data were obtained from the Longitudinal Integration Database for Health and Labour Market Studies (LISA) [57].

*Family income* was used as an alternative indicator of socioeconomic position (data from LISA database). It measures the total earned income for both the father and the mother from the year of the index person’s birth to the year of their compulsory school graduation. The total earned income for each year was summed and then averaged over the years (16) since the index person’s birth. Index persons were grouped into quartiles based on the income variable, using a similar approach to that applied for the parental education variable.

### Covariates

Four covariates potentially related to mental disorder and academic achievement were included: *maternal country of birth* (Sweden or outside of Sweden; data obtained from the Medical Birth Register where only maternal country of birth is registered); *family type* (whether the child lived with both biological parents in the year of compulsory school graduation or not; Total Population Register); *number of siblings* (Total Population Register), and *maternal* and *paternal psychiatric morbidity.* The latter two covariates use data from the NPR and indicate (no/yes) whether the corresponding parent had been hospitalised with a main diagnosis of mental disorder (ICD-9: 290–319, ICD-10: F00-F99) since the birth of the index person. *Gender* (boy/girl) was used as both a covariate and a stratification variable (data from the Total Population Register).

### Statistical analyses

Analysis of variance (ANOVA) and *t*-tests were used to examine mean differences in the main variables of the study between boys and girls in different socioeconomic positions. Bivariate and multivariate linear regression were used to investigate associations between the indicators of mental disorder and grade points. Outcomes were analysed using four models. Model 1a reflects the association between psychiatric hospitalisation events and school grades only. Model 1b is adjusted for all the covariates. Model 2a shows the association between psychoanaleptic drug prescriptions and school grades, and model 2b is adjusted for all the covariates. Because we examine complete cohorts rather than a sample of the total population, *p*-values and standard errors are superfluous and not presented. Instead, beta coefficients of *z*-scores of grade points with upper and lower 95% confidence intervals are presented. Interaction analyses were performed using the fully adjusted models (model 1b and model 2b) to compare the effects of mental disorders on grade points between children in different social groups and between boys and girls within these groups. Stratified analyses were performed when interaction effects of *p* < 0.05 were identified. All analyses were performed in Stata v. 14.

## Results

Table 1 shows a stepwise social gradient in mental disorders where mean number of psychiatric hospitalisation events as well as proportions of prescribed psychoanaleptic medication decreased by increased parental level of education and income. Similarly, there was a clear gradient in academic achievement (not shown in table). A one-way ANOVA was performed to compare the mean grade points between the four groups of parental level of education (compulsory, *M* = − 0.62, SD = 1.06, 2 year upper secondary, *M* = − 0.279, SD = 0.930, 3 year secondary, *M* = 0.034, SD = .90, post-secondary, *M* = 0.391, SD = 0.872), which revealed a statistically significant difference (*F* (3, 579,851) = 27,146.40, *p* < 0.001, η^2^ = 12.3. Thus, 12.3% of the total variance in academic achievement was accounted for by group membership (results from the Tukey post hoc test are available in Additional file 1). Given the benchmarks provided by Cohen (1988) to define small (η^2^ = 0.01), medium (η^2^ = 0.06), and large (η^2^ = 0.14) effects, these differences are quite large.

**Table 1:** Population characteristics and mental disorder indicators related to study variables

	All		Mental disorder indicators	
	N	% of total	Psychiatric hospitalisation events, m (s.d.)	Psychoanaleptics prescription, %
Total	643 558	100.00	0.023 (0.25)	6.19
Sex				
Male	330 667	51.38	0.019 (0.24)	5.56
Female	312 891	48.62	0.028 (0.26)	6.86
Parental level of education				
Compulsory	39 296	6.57	0.038 (0.29)	8.67
Upper secondary two year	212 933	35.61	0.027 (0.30)	7.58
Upper secondary three year	98 437	16.46	0.021 (0.20)	5.58
Post-secondary	247 257	41.35	0.021 (0.23)	5.48
Missing (% of total)	45 635	13.10	0.012 (0.16)	2.76
Family income (quartiles)				
1^st^ quartile	160 893	25.00	0.026 (0.24)	6.29
2^nd^ quartile	160 886	25.00	0.024 (0.21)	7.42
3^rd^ quartile	160 890	25.00	0.021 (0.31)	5.92
4^th^ quartile	160 888	25.00	0.021 (0.23)	5.37
Family Type				
Both biological parents	450 588	70.01	0.019 (0.20)	5.39
Not both biological parents	161 996	25.17	0.039 (0.37)	9.22
Missing (% of total)	30 974	4.82	0.004 (0.06)	2.03
Number of siblings				
0	38 284	5.95	0.022 (0.23)	5.58
1	229 292	35.63	0.020 (0.28)	5.44
2	193 258	30.00	0.021 (0.21)	5.89
3	96 321	14.50	0.025 (0.23)	6.83
4	45 100	7.00	0.033 (0.26)	8.04
5	21 243	3.30	0.035 (0.31)	8.84
6 +	20 060	3.12	0.039 (0.30)	8.77
Mother’s birth country				
Sweden	496 470	77.15	0.024 (0.26)	6.69
Not Sweden	70 535	10.96	0.027 (0.24)	5.17
Missing (% of total)	76 553	11.90	0.017 (0.19)	3.93
Maternal psychiatric morbidity				
Yes	24 727	3.84	0.064 (0.39)	13.62
Paternal psychiatric morbidity				
Yes	24 962	3.88	0.048 (0.29)	12.33

A second one-way ANOVA was performed to compare the mean grade points between the 1st (M = − 0.361, SD = 1.14), 2nd (M = − 0.138, SD = 0.965), 3rd (M = 0.048, SD = 0.879), and 4th (M = 0.395, SD = 0.861) income quartile, which also showed a statistically significant difference (F (3, 605,457) = 16,371.32, *p* < 0.001, h2 = 0.075. Here, 7.5% of the total variance in academic achievement was accounted for by group membership, indicating a medium-sized effect. These results show that the variance in academic achievement to a larger degree is explained by parental level of education (12.3%) than family income (7.5%).

Additionally, girls obtained higher grade point scores (*M* = 0.168, *SD* = 0.001) than boys (*M* = − 0.162, *SD* = 0.001), *t* (605459) = − 1.3, *p* < .001, *d* = 0.33). The two indicators of mental disorders were slightly more common in girls than in boys; girls (*M* = 0.028, SD = 0.26) had more days hospitalised than boys (*M* = 0.019, *SD* = 0.24), *t* (643555) = − 20.3, *p* < 0.001, *d* = 0.36 and a higher frequency of psychoanaleptic drug prescription χ^2^(1, *N* = 643,557) = 451.1, *p* < 0.001, *d* = 0.05. While the differences in grade point scores and hospitalisation events were small to medium-sized, the differences in psychoanaleptic drug prescription were very small [58] .

The regression analyses show that both indicators of mental disorder were associated with reduced academic achievement. The unadjusted and adjusted Beta coefficients for psychiatric hospitalisation were − 0.36 (CI = − 0.38, 0.35) and − 0.33 (CI = -0.35, − 0.33) respectively. In other words, each hospitalisation event due to a mental disorder was associated with a decrease in grade points of 0.33 standard deviations when taking a range of possible confounders into account. The corresponding coefficients for being prescribed psychoanaleptics were − 0.75 (CI = − 0.76, −-0.74) and − 0.70 (CI = − 0.71, −-0.69).

Given the identified gender pattern in both the predictors and the outcome, stratified analyses by gender and SEP were performed. As shown in Tables 2 and 3, mental disorder was more strongly associated with reduced grades among boys than among girls regardless of SEP category. The negative association between psychiatric hospitalisation and grade points was strongest among boys in the highest SEP. Among girls, the association was strongest in the second highest SEP.

**Table 2:** Associations between psychiatric disorder indicators and academic achievement by parental level of education and gender. Beta coefficients of *z*-scores of grade points (with upper and lower 95% confidence intervals in parenthesis). Adjusted for all covariates

	Compulsory		2 year upper sec.		3 year upper sec.		Post-secondary	
	Boys	Girls	Boys	Girls	Boys	Girls	Boys	Girls
	Beta (CI)	Beta (CI)	Beta (CI)	Beta (CI)	Beta (CI)	Beta (CI)	Beta (CI)	Beta (CI)
Psychiatric hospitalisations	–0.42 (–0.51, –0.33)	–0.33 (–0.37, –0.27)	–0.39 (–0.42, –0.36)	–0.34 (–0.36, –0.31)	–0.43 (–0.48, –0.37)	–0.40 (–0.44, –0.36)	–0.47 (–0.51, –0.43)	–0.25 (–0.27, –0.23)
Psychoanaleptics prescription	–0.69 (–0.75, –0.63)	–0.62 (–0.76, –0.56)	–0.78 (–0.80, –0.76)	–0.65 (–0.67, –0.63)	–0.79 (–0.83, –0.76)	–0.71 (–0.74, –0.68)	–0.75 (–0.77, –0.72)	–0.59 (–0.61, –0.57)
	*N* = 16 251	*N* = 15 433	*N* = 101 818	*N* = 97 682	*N* = 45 349	*N* = 43 573	*N* = 116 435	*N* = 111 085

**Table 3:** Associations between psychiatric disorder indicators and academic achievement by family income quartiles and gender. Beta coefficients of *z*-scores of grade points (with upper and lower 95% confidence intervals in parenthesis). Adjusted for all covariates

	1^st^ quartile		2^nd^ quartile		3^rd^ quartile		4^th^ quartile	
	Boys	Girls	Boys	Girls	Boys	Girls	Boys	Girls
	Beta (CI)	Beta (CI)	Beta (CI)	Beta (CI)	Beta (CI)	Beta (CI)	Beta (CI)	Beta (CI)
Psychiatric hospitalisations	–0.34 (–0.39, –0.29)	–0.31 (–0.35, –0.28)	–0.43 (–0.48, –0.39)	–0.40 (–0.36, –0.31)	–0.51 (–0.55, –0.46)	–0.26 (–0.28, –0.23)	–0.46 (–0.51, –0.43)	–0.26 (–0.29, –0.24)
Psychoanaleptics prescription	–0.79 (–0.83, –0.75)	–0.73 (–0.77, –0.69)	–0.82 (–0.85, –0.80)	–0.68 (–0.71, –0.66)	–0.78 (–0.83, –0.76)	–0.64 (–0.66, –0.62)	–0.72 (–0.75, –0.69)	–0.56 (–0.59, –0.54)
	*N* = 43 037	*N* = 41 289	*N* = 78 627	*N* = 75 218	*N* = 79 949	*N* = 76 112	*N* = 79 318	*N* = 76 165

Models adjusted for all covariates

To further explore these gender and SEP patterns, interaction analyses were performed using the highest SEP categories as reference. As shown in Table 4 and 5, the interaction effects of SEP and psychiatric hospitalisation were more pronounced among girls than among boys. The grade points of girls in the mid SEP groups were particularly negatively affected by psychiatric hospitalisation. For example, hospitalisation events among girls whose parents had completed 3 years of upper secondary education was associated with − 0.15 standard deviations in grade points per event, compared to those with parents with a post-secondary education. The corresponding result for girls in the second family income quartile was –-0.13. Table 4 further shows that this U-shaped pattern among girls also was detected for psychoanaleptic medication prescription and the SEP indicator of parental level of education. No interactions were identified among boys with regards to psychoanaleptic medication prescription. Worth noting are the statistically significant positive coefficients among boys. Compared to their high SEP peers, psychiatric hospitalisation was associated with an increase in grade points by 0.08 standard deviations per each hospitalisation event for boys in the two-year upper secondary parental education category. Among boys in the lowest family income quartile, the grade points improved by 0.13 standard deviations per each hospitalisation event. The interaction analyses for family income and prescription of psychoanaleptics showed a linear pattern; the interaction decreased by increasing level of income, primarily among girls (Table 5).

**Table 4:** Models displaying interactions between mental disorder indicators and parents’ level of education for boys and girls. Beta coefficients of *z*-scores of grade points (with upper and lower 95% confidence intervals in parenthesis)

	Boys		Girls	
	Model 1a Beta (CI)	Model 1b Beta (CI)	Model 2a Beta (CI)	Model 2b Beta (CI)
Parental level of education				
Compulsory	–0.85 (–0.87, –0.84)	–0.85 (–0.86, –0.83)	–0.89 (–0.90, –0.87)	–0.87 (–0,88, –0.85)
Upper secondary two year	–0.62 (–0.63, –0.61)	–0.60 (–0.61, –0.60)	–0.62 (–0.63, –0.62)	–0.61 (–0.62, –0.31)
Upper secondary three year	–0.34 (–0.35, –0.33)	–0.33 (–0.34, –0.32)	–0.33 (–0.34, –0.32)	–0.32 (–0.33, –0.31)
Post-secondary (Ref.)	0	0	0	0
Psychiatric hospitalisations	–0.43 (–0.50, –0.36)		–0.25 (–0.27, –0.23)	
× Compulsory	0.04 (–0.05, 0.12)		–0.10 (–0.15, –0.04)	
× Upper secondary two year	0.08 (0.03, 0.13)		–0.09 (–0.12, –0.06)	
× Upper secondary three year	0.04 (–0.30, 0.11)		–0.15 (–0.20, –0.11)	
× Post-secondary (Ref.)	0		0	
Psychoanaleptics prescription		–0.75 (–0,77, –0.73)		–0.59 (–0.61, –0.56)
× Compulsory		0.03 (–0.02, 0.08)		–0.09 (–0.14, –0.04)
× Upper secondary two year		–0.04 (–0.07, –0.00)		–0.08 (–0.11, –0.05)
× Upper secondary three year		–0.04 (–0.08, 0.00)		–0.12 (–0.16, –0.08)
× Post-secondary (Ref.)		0		0
N	279 853		267 772	

Confidence intervals that do not include 0 indicate that estimates are statistically significant at the 5 % level (*p* < 0.05). Model 1a-1b displays the results for *boys*, model 2a-2b for *girls*. Models adjusted for all covariates

**Table 5:** Models displaying interactions between psychiatric disorder indicators and *family income quartiles* for boys and girls. Beta coefficients of *z*-scores of grade points (with upper and lower 95% confidence intervals in parenthesis)

	Boys		Girls	
	Model 1a Beta (CI)	Model 1b Beta (CI)	Model 2a Beta (CI)	Model 2b Beta (CI)
Family income				
1^st^ quartile	–0.32 (–0.33, –0.31)	–0.31 (–0.32, –0.30)	–0.31 (–0.32, –0.30)	–0.30 (–0,31, –0.28)
2^nd^ quartile	–0.26 (–0.27, –0.25)	–0.25 (–0.26, –0.24)	–0.25 (–0.26, –0.24)	–0.24 (–0.25, –0.23)
3^rd^ quartile	–0.18 (–0.19, –0.17)	–0.17 (–0.18, –0.16)	–0.16 (–0.17, –0.15)	–0.15 (–0.16, –0.15)
4^th^ quartile (Ref.)	0	0	0	0
Psychiatric hospitalisations	–0.46 (–0.51, –0.41)		–0.26 (–0.28, –0.23)	
× 1^st^ quartile	0.13 (0.06, 0.20)		–0.06 (–0.10, –0.02)	
× 2^nd^ quartile	0.04 (–0.03, 0.10)		–0.13 (–0.17, –0.09)	
× 3^rd^ quartile	–0.02 (–0.10, 0.04)		0.00 (–0.03, 0.04)	
× 4^th^ quartile (Ref.)	0		0	
Psychoanaleptics prescription		–0.70 (–0,77, –0.73)		–0.55 (–0.57, –0.52)
× 1^st^ quartile		–0.08 (–0.12, –0.04)		–0.18 (–0.22, –0.14)
× 2^nd^ quartile		–0.10 (–0.14, –0.06)		–0.12 (–0.15, –0.08)
× 3^rd^ quartile		–0.05 (–0.09, –0.01)		–0.07 (–0.11, –0.04)
× 4^th^ quartile (Ref.)		0		0
N	279 853		267 772	

Confidence intervals that do not include 0 indicate that estimates are statistically significant at the 5 % level (*p* < 0.05). Model 1a-1b displays the results for *boys*, model 2a-2b for *girls*. Models adjusted for all covariates

## Discussion

This study aimed to investigate social gradients in the association between mental disorder in childhood/adolescence and academic achievement at 15–16 years of age. Both indicators of mental disorder (hospitalisation due to a psychiatric diagnosis and prescription of psychoanaleptics) were associated with lower grade points in all SEP groups. These findings confirm previous results [5, 17, 20, 21]. Interaction analyses showed that level of parental education primarily moderated the association between mental disorder and academic achievement among girls. This is consistent with the conclusions of Brännlund and Edlund (2020) although the present study analysed the social gradient more closely than was done in their work, for example by using a four-category measure of parental education rather than two-categories, and also family income. Girls whose parents had completed 3 years of upper secondary education or belonged to the second family income quartile experience the most severe adverse effects on school grades due to mental disorder. However, it should be noted that boys’ grades were by no means unaffected by previous mental disorder. In contrast to several other studies [17, 19, 37, 38], our study showed stronger negative associations between mental disorder and grade points in boys than in girls. However, there appears to be no typical social gradient in this association among boys although some findings deserve attention, which will be discussed below.

Based on previous findings [44, 45] and theory on the importance of access to cultural and economic resources [56], we expected that both boys and girls in the lowest SEP categories would exhibit the strongest adverse effects on school grades due to mental disorder, and that severity would decline as level of parental education and income increased. Instead, the findings indicate a U-shaped pattern among girls and no or inverse gradient in boys. Several interpretations of our findings are possible. First, low SEP families might experience elevated and varying types of stressors, combined with greater barriers to accessing resources relative to other groups; this is likely to affect both mental health and school achievement [59, 60]. For example, because children of low SEP parents already have the lowest level of achievement of the four groups, it is possible that they do not have much further to fall even when exposed to adverse circumstances. This potential ‘floor effect’ was identified in a European study on the educational consequences of parental divorce; divorce does not worsen the school outcomes of children from poorly educated families because they already have limited educational opportunities [61].

Second, it is possible that children and adolescents with poor mental health from less educated families actually benefit more from hospital admission and medicine prescription than their more advantaged peers do. School success has, for example, been identified as a positive effect of mental health medication [62]. The unexpected results for boys with regard to indications of improved grade points due to hospitalisation for the 2nd categories of both SEP measures, might reflect this. However, more research is required to understand this interaction further and to determine whether this effect is equally strong in all socioeconomic groups.

Third, school-related stress may help explain the identified drop in academic achievement among girls of parents with intermediate levels of education. It has been suggested that school-related demands explain the higher incidence of poor mental health in adolescent girls compared to boys [38]. It has also been established that while school-related stress is prevalent in youths from mid/high socioeconomic backgrounds (especially girls), those from low socioeconomic backgrounds are more stressed about relationships and lack of money [63–66]. There might also be an incongruence between ambition and available resources for girls in the mid SEP categories. In other words, they and their parents might experience pressure and have high ambitions relating to school performance, but lack familial resources (i.e. cultural and economic capital) to “buffer” the negative impact of mental disorder on the child’s school achievement [56, 63, 67]. Such “buffers” may include familial or social academic support networks, private tutors, or other privately financed educational interventions [67]. A similar argument would be probable for income categories; a child whose parents have high income but relatively little experience of studying will probably have other kinds of academic socialisation and adopt different study strategies to a child whose parents are more highly educated but less well-paid, leading to differences in school grades.

Although level of education typically represents access to cultural capital relevant to academic achievement, educational outcomes must be considered *in combination* with resources such as income [56]. These SEP indicators are typically highly correlated. However, the difference between parental education and family income can be explained by supposing that parental education represents a type of cultural capital necessary for children’s academic achievement that extends over and beyond income. For example, blue collar professions do not require extensive theoretical study but can nevertheless be well-paid, while other occupations that require longer educations provide relatively low wages [68].

With regard to the gender patterns identified in this study, it is noteworthy, that despite Sweden’s high international rankings with regard to gender equality, income levels and the labour market are strongly gendered in Sweden; on a group level, women are disadvantaged relative to men [69]. It is likely that these circumstances influence the aspirations of girls and boys. Although girls receive better grade points than boys do, achieving good grades may be seen as a more important route to future financial security and labour market opportunities for girls than for boys. This might lead to greater stress about school performance among girls from mid SEP backgrounds even if they generally show better school results than their male peers do.

The strengths of this study include its use of high quality data and a large sample size, which enable broad generalisation. The use of register data reduces the risk of recall and selection bias. However, some limitations deserve attention. First, the chosen indicators of mental disorder: psychiatric hospitalisation is rare and is a rather crude indicator because it can result from a wide range of disparate diagnoses including drug abuse, depression, and schizoaffective disorders. Prescription of mental health medication is more common but is still a relatively imprecise measure of poor mental health. Our conclusions therefore relate to mental disorder in its broadest sense. We acknowledge that other types of mental health problems are not captured by these indicators and that we cannot distinguish which disorder, if any, that is most strongly related to academic achievement. Therefore, given the limitations of hospitalisation and medication as indicators of mental disorders, and that many children might never receive the appropriate attention and/or diagnosis, future studies should differentiate between types of diagnoses and also include symptomatology. Second, the categorisation of family income based on quartiles likely does not capture the same people as within the education group. Hence, these are not directly comparable.

## Conclusions

This study suggests that, in contrast to an expected linear social gradient, there is a U-shaped social gradient in the relationship between mental disorder in childhood or adolescence and grade points at age 15/16 years, especially among girls. Specifically, the school grades of girls from families of intermediate socioeconomic position were more strongly affected by mental disorder (as indicated by hospitalisation and prescription of psychoanaleptic medication) than those of their peers in the low and high SEP categories. This study shows the importance of recognising several indicators of socioeconomic position as well as gender when investigating links between mental health and academic achievement. Future research should address both socioeconomic background and gender disparities to clarify the links between mental health and school-related outcomes in children and adolescents.

## Supplementary Information

**Additional file 1.**

## Data Availability

Data is not publicly available. Please contact Umeå SimSam Lab for further information on data availability.

## Abbreviations

ADHD: Attention-deficit/hyperactivity disorder
ATC: Anatomical therapeutic chemical classification system
ICD: International classification of disease
LISA: Longitudinal integration database for health and labour market studies
NPR: Swedish national patient register
PDR: Swedish prescribed drug register
SEP: Socioeconomic position
