# Correction to: Is there a social gradient in how youth with mental disorder perform academically? Findings from a Swedish longitudinal register-based study

**DOI:** 10.1186/s12888-021-03465-y

**Published:** 2021-09-17

**Authors:** Evelina Landstedt, Cristian Bortes, Mattias Strandh

**Affiliations:** 1 Department of Social and Psychological Studies, Karlstad University, Universitetsgatan 2, SE-651 88 Karlstad, Sweden; 2 Department of Social Work, Umeå University, SE-907 87 Umeå, Sweden; 3 Centre for Research on Child and Adolescent Mental Health, Karlstad University, SE-651 88 Karlstad, Sweden

**Correction to: BMC Psychiatry 21, 441 (2021)**

**https://doi.org/10.1186/s12888-021-03448-z**

Following publication of the original article [1], the author reported that the Abstract was missing in the published article.

The original article [1] has been corrected.
