# Supplementary material for: Is there a social gradient in how youth with mental disorder perform academically? Findings from a Swedish longitudinal register-based study
Source: BMC Psychiatry. 2021 Sep 6;21:441. doi: 10.1186/s12888-021-03448-z (PMC8422624; doi:10.1186/s12888-021-03448-z)
Supplement: Supplementary file 1 — Additional file 1. [file 12888_2021_3448_MOESM1_ESM.docx]

|  |  |  | Tukey | | |
| --- | --- | --- | --- | --- | --- |
| z-standardised grade points | Contrast | Std. Err. | *t* | *P* < | 95 % CI |
| 1 vs. 2 | 0.34 | 0.005 | 66.17 | 0.001 | 0.33, 0.35 |
| 1 vs. 3 | 0.65 | 0.006 | 117.08 | 0.001 | 0.64, 0.67 |
| 1 vs. 4 | 1.02 | 0.005 | 198.74 | 0.001 | 1.00, 1.03 |
| 3 vs. 2 | 0.31 | 0.003 | 88.12 | 0.001 | 0.30, 0.32 |
| 4 vs. 2 | 0.67 | 0.003 | 247.19 | 0.001 | 0.67, 0.68 |
| 4 vs. 3 | 0.36 | 0.003 | 104.09 | 0.001 | 0.35, 0.37 |

**Appendix**

Results from Tukey post hoc test, comparing academic achievement between the four socioeconomic groups

1= Compulsory education, 2= Two year upper secondary education, 3= Three year upper secondary education, 4= Tertiary education
